# Draft Genome Sequence of the Toluene-Degrading, Dissimilatory Sulfate-Reducing Bacterium *Desulforhabdus* sp. Strain TSK

**DOI:** 10.1128/mra.00295-22

**Published:** 2022-08-11

**Authors:** Reina Tsukuda, Taketo Sugimoto, Kazuhiro Takamizawa, Kohei Nakamura

**Affiliations:** a The Graduate School of Natural Sciences and Technologies, Gifu University, Gifu, Japan; b Faculty of Applied Biological Sciences, Gifu University, Gifu, Japan; Montana State University

## Abstract

The draft genome sequence of *Desulforhabdus* sp. strain TSK, which oxidizes toluene under dissimilatory sulfate-reducing conditions, had an estimated size of 4,933,642 bp.

## ANNOUNCEMENT

Brine in oil reservoirs and coextracted seawater during oil mining is sulfate-rich. Dissimilatory sulfate-reducing bacteria (DSRB) present in such reservoirs pose problems to the petroleum industry, because the resulting sulfide lowers oil and natural gas quality and causes iron corrosion in transport pipes (1). The crude oil components benzene and toluene are potential electron donors for sulfate-reducing bacteria (2). Research on the biochemical and molecular ecological properties of such aromatic hydrocarbon-degrading DSRB is crucial for reducing the petroleum industry’s economic losses. We isolated a toluene-degrading DSRB, strain TSK, from a crude oil-producing pond and reported its draft genome sequence.

One-liter medium bottles were filled with water and sediment from the crude oil-producing pond in Myohoji (Niigata, Japan), closed with screw caps, and stored at 4°C until analysis. The strain TSK was enriched with the sediment using a basal medium (3) supplemented with toluene and sulfate and then was isolated from colonies formed in anaerobic tubes containing basal medium comprising either toluene (0.2 mM) or acetate (10 mM), FeSO_4_ (10 mM), yeast extract (0.01%), and Na_2_S·9H_2_O (0.5 mM) and solidified with gellan gum (0.1%). Genomic DNA was extracted from a single colony and a culture grown in liquid medium with acetate and FeSO_4_, using the MagList 5M genomic DNA extraction kit (Bioneer Corp., Republic of Korea) and a conventional method (4), respectively. Libraries for the DNA extracted from the two sources were individually constructed using the Illumina DNA preparation kit, following the manufacturer’s protocol. The libraries were sequenced using an Illumina MiSeq system, and 3.5 million paired-end reads were obtained in 2 × 300-bp format. Default parameters were used for all software unless stated otherwise. Using Fastp v0.21.0 (5), reads from both libraries were trimmed for adaptor sequences and low-quality bases (Q scores of <30), providing 3.3 million reads (663 Mb). Quality-filtered paired-end and unpaired reads were assembled using SPAdes v3.15.2 with isolate and k-mer size (21, 33, 55, 77, 99, and 127) options (6), and contigs with <200-bp lengths were discarded. Assembly statistics were computed using QUAST v5.0.2 (7). The assembly consisted of 300 contigs with a total sequence length of 4,933,642 bp, with the largest contig having 295,967 bp, an *N*_50_ value of 112,314 bp, a GC content of 56.1%, and coverage of 139×. Genome annotation was primarily performed with programs implemented in the DFAST pipeline (8), such as Prodigal v2.6.3 (9), ARAGORN v1.2.38 (10), and CRT v1.2 (11). Furthermore, rRNA genes were predicted using Barrnap v0.9 (12). Some genes involved in anaerobic toluene degradation were annotated using Prokka v1.14.6 (13). The draft genome contained 4,342 coding sequences (CDSs), 10 rRNAs, including 6 partial rRNAs, 49 tRNAs, 1 transfer-messenger RNA, and 5 CRISPRs.

Phylogenetic relatedness computed using an application (16S-based ID) in EzBioCloud (14) was observed between strain TSK (DSTSK_R00060) and strain PRTOL1 (GenBank accession number U49429) and Desulforhabdus amnigena ASRB1^T^ (GenBank accession number X83274), with 99.7% and 97.8% similarities, respectively. The most related strains were Desulfoglaeba alkanexedens ALDC (GCA_005377625.1) and Desulfacinum hydrothermale DSM 13146 (GCA_900176285.1), with average nucleotide identity (ANI) values of 76.43% and 76.18%, respectively, determined using DFAST.

Genes and gene clusters for anaerobic toluene degradation, including the *bss* cluster encoding benzylsuccinate synthase and a set of genes (*bamBC*, *dch*, *had*, and *bamA*) encoding enzymes for benzoyl-coenzyme A (CoA) degradation, were detected in the genome of strain TSK.

The information obtained from the genome of strain TSK may provide new molecular and ecological insights into aromatic hydrocarbon-degrading DSRB in oil reservoirs. The strain TSK can be obtained from the authors upon request.

### Data availability.

The draft genome sequence of *Desulforhabdus* sp. strain TSK has been deposited in DDBJ/ENA/GenBank under the accession numbers BQXM01000001 to BQXM01000300. The version described in this paper is the first version, BQXM00000000.1. Raw sequence reads were deposited in the Sequence Read Archive (SRA) with accession numbers DRR353549 and DRR353550 under BioSample accession number SAMD00446890 and BioProject accession number PRJDB13207.
